# Lost to follow up from chronic care services during COVID-19 from health facilities, in Northwest Ethiopia

**DOI:** 10.3389/fepid.2022.883316

**Published:** 2022-11-25

**Authors:** Daniel Gashaneh Belay, Asmamaw Adugna

**Affiliations:** ^1^Department of Human Anatomy, College of Medicine and Health Sciences, University of Gondar, Gondar, Ethiopia; ^2^Department of Epidemiology and Biostatistics, College of Medicine and Health Sciences, Institute of Public Health, University of Gondar, Gondar, Ethiopia; ^3^Department of Health Education and Behavioral Sciences, College of Medicine and Health Sciences, University of Gondar, Gondar, Ethiopia

**Keywords:** lost to follow up, COVID-19, HIV/AIDS, hypertension, Ethiopia

## Abstract

**Introduction:**

The COVID-19 pandemic and the responses to it have greatly altered individual lives, particularly those with chronic illnesses. The pandemic affected the processes of routine comprehensive care for patients. Because chronic illnesses depress the immune system, they make individuals more susceptible to infection as well as more sickly and less likely to recover from the COVID-19 pandemic. Because of this, the rate of loss to follow-up (LTFU) from chronic illness care is accelerated by the COVID-19 pandemic, and the number of patients receiving new treatments is decreased. Therefore this study aimed to assess the mean difference of loss to follow-up among HIV/AIDS, diabetes mellitus (DM), and hypertension patients during the COVID-19 period as compared to pre-COVID-19 at health facilities in Northwest Ethiopia.

**Methods:**

An institution-based secondary data analysis of the Gondar city health report from October 2019 to Jun 2021 was done. Excel data were transformed to STATA 14 for analysis. An independent *t*-test was used to compare the mean difference of loss to follow-up and new initiation of treatment among HIV/AIDS, DM, and hypertension patients during the pre-COVID-19 and COVID-19 periods at facilities in Northwest Ethiopia. Variables with a mean difference of *p* < 0.005 with a 95% confidence interval were used to declare the significant level.

**Result:**

There was a significantly higher mean difference in the number of patients with LTFU from Anti-Retroviral Therapy (ART) and antihypertensive treatment during COVID-19 as compared to the pre-COVID-19 period [μd =17.85, 95%CI: 3.25, 32.95] and [μd =17.31, 95%CI: 8.35, 26.97] respectively. The mean number of patients who were newly started anti-hypertensive treatment during the COVID-19 season was significantly decreased as compared to those who were before the COVID-19 period [μd = −32.94, 95%CI: −63.76, −2.12].

**Conclusion:**

There was a significantly higher mean difference in the number of patients with LTFU from Anti-Retroviral Therapy (ART) and antihypertensive treatment during COVID-19 as compared to the pre-COVID-19 period. The mean number of patients who were newly started anti-hypertensive treatment during the COVID-19 season was significantly decreased as compared to the pre-COVID-19 period. Therefore the Ministry of Health Ethiopia (MOH) should update and prepare convenient care and follow-up such as remote chronic illness management methods during the ongoing COVID-19 pandemic in Ethiopia.

## Background

Coronavirus disease 2019 (COVID-19) is an infectious disease caused by a newly discovered coronavirus which is labeled as SARS-CoV-2 (1). Ethiopia confirmed its first case of COVID-19 on 13 March 2020, 2 days later the WHO declared a pandemic of the disease (2). In Ethiopia, COVID-19 measures were adopted on 16 March and a 5-month state of emergency was declared on April 10, when COVID-19 becomes sporadic (3).

The COVID-19 pandemic and the responses to it have greatly altered individual lives, particularly those with chronic illnesses (4, 5). Chronic diseases such as; human immuno virus (HIV), diabetes mellitus (DM), and hypertension are immunosuppressing cases making patients not only more vulnerable to infections but also makes more likely to have severe illnesses from COVID-19 (6–9). Moreover, these patients with COVID-19 are less likely to be cured (8). For example, deaths due to HIV over 5 years could increase by up to 10%, as compared with the pre-COVID-19 pandemic in high-burden settings (9).

The pandemic affected the processes of routine comprehensive care for chronic patients due to disrupted delivery care in different ways (8, 10). Firstly, the implementation of lockdowns, border closures, quarantine, social distancing, and community containment measures are impacting the availability, and accessibility of medications (4, 10, 11). Second, hospitals were busy because they are preoccupied with COVID-19 patients, which led to patients who needed chronic follow-up to postpone their follow-up (8, 10, 11). Therefore, the pandemic fuels panic especially in sub-Saharan Africa where the healthcare system is fragile in withstanding the disease (2).

Due to the physical distancing required to combat COVID-19 which increases loneliness, an individual with chronic health conditions with a compromised immune system may develop a stronger stress response to COVID-19 than the rest of the population (4). As a result, they are obligated to lose follow-up from chronic disease care (10, 12). Public health responses toward COVID-19 are stressful to PLWHs (Patient living with HIV's) and disrupt their access to ART (12). Studies showed that about 25% of individuals in ART interrupted their medication during COVID-19 responses period (5). In India since the estimated final cost of exported antiretroviral medicines increased from pre-COVID-19 prices, PLWHs on ART are obligated to interrupt their treatment (4, 11). A study in Turkey showed that there was a worsening in lifestyle parameters of DM patients during the COVID-19 lockdown (13). Another study in Australia showed that patients with hypertension reported greater anxiety as compared to the controls (14). But no study has shown the magnitude of loss to follow-up from chronic care during COVID-19 in Ethiopia. Therefore this study aims to assess the disruptions in chronic disease care among HIV/AIDS, DM, and hypertension patients caused by the COVID-19 pandemic at health facilities in Northwest Ethiopia.

## Methods

### Study design, setting, and period

Institution-based secondary data analysis was conducted in Gondar city from October 2019 to Jun 2021. Gondar city is located about 727 km away from Addis Ababa, the capital city of Ethiopia, and 180 km away from Bahir Dar, the capital city of Amhara Regional State. The city has a total 338, 646 population, of which 256,041 are between 18 and 65 years old. Gondar has 6 sub-cities and 27 Kebele with a total of 78,772 households. There are 9 public health institutions delivering chronic care (8 health centers and 1 specialized hospital) and more than 40 private health facilities in the city administration. About 41,000 clients visited the 3 chronic care service units in all 9 health institutions.

In Ethiopia, COVID-19 measures were adopted on March 16 and further improved on March 20. Then, on April 10, a 5-month state of emergency including lockdown was declared (3). Therefore, the pre-COVID period was a period before March 2020**;** the COVID-19 period was a period from March 2020 to Jun 2021.

### Study participants and data source

All patients with HIV/AIDS, and/or diabetes mellitus (DM), and/or hypertension, and follow-up care services in Gondar city public health facilities during the study period were included in this study. The Demographic and Health Information System (DHIS-2) system central data repository of North West Ethiopia contains data from all 9 health facilities for chronic care management. All health facilities were replicating their data to the central repository; this ensured that the central data mirrored the data available at the sites through the full data recording period. Data were extracted from the central repository in December 2021. Excel data were transformed to STATA 14 for analysis.

### Outcomes of interest

The study included patient data aggregated in monthly values at the health facility level. Our study sought to assess the loss of follow-up and the new treatment initiation among HIV/AIDS, diabetes mellitus, and hypertension patients before and after the confirmation of the first cases of COVID-19 in Ethiopia on March 19, 2020, which used as a starting mark of “COVID-19 period”.

### Operational definitions

#### Chronic care services

Care services include HIV/AIDS, DM, and hypertension.

#### Hypertension

This is when systolic blood pressure is ≥140 mmHg and/or a diastolic pressure of ≥90 mmHg.

#### Treatment regimen

This is based on the WHO national consolidated guidelines for comprehensive HIV prevention, care, and treatment in 2018 (15).

#### New treatment initiation

A patient newly started treatment for HIV/AIDS, DM, and/or Hypertension.

#### Lost to follow-up

A patient didn't take their ART and/or antihypertensive and/or DM treatment medications for at least 1 month and above.

### Data management and analysis

Data cleaning, recoding, and transforming were done using Excel and exported to Stata 14 software for further statistical analyses. For the missing data, the imputation technique was done by filling it to create a complete data matrix to analyze using the standard method without defining an explicit model for it. Therefore multiple imputation techniques were used for the three outcome variables (HIV/AIDS, hypertension, and DM) because they had only < 5% missing data in the outcomes. Descriptive statistics, counts, means, and tables and graphs were used to describe the characteristics of the study participants and present the study results. To compare the mean difference in patients with HIV/AIDS, diabetes, and hypertension who were lost to follow-up and those who started new treatments during the pre-COVID-19 and COVID-19 periods, an independent *t*-test was conducted. Variables with a mean difference of *p* < 0.005 with a 95% confidence interval were used to declare the significant level.

### Ethical consideration

The institutional review board (IRB) of the University of Gondar approved the research proposal and gave ethical clearance reference number V/P/RCS/05/574/2020. On the other side, permission and support letters were obtained from the Gondar city health office. However, the study used only de-identified secondary data, and no investigators interacted with human subjects or had access to identifiable data or specimens for research purposes, so a consent waiver was obtained for the study.

## Results

### HIV/AIDS, DM, and hypertension patients among health facilities in Northwest Ethiopia

A total of 10,356 DM, 12,744 HTN, and 296,593 HIV/AIDS patients from nine health facilities were reported within 21 months. Of the total DM patients, most of the study participants (63.99%) were Type II DM and only a few percent (0.25%) of the participants were Gestational Diabetes mellitus (GDM). Of the total hypertensive patients, more than three-fifths (67.03%) were stage I, and 12.32% were severe hypertension. Of all HIV/AIDS patients, three-quarters (75.05%) were adults in 20–49 years old (Table 1).

**Table 1:** The number of HIV/AIDS, DM, and hypertension patients among health facilities in Northwest Ethiopia from October 2020 to Jun 2021.

**Chronic disease type**	**Categories**		**Frequency (*n*)**	**Percent (%)**	**Total**
DM	Type I		3,703	35.76	10,356
	Type II		6627	63.99	
	Gestational Diabetes mellitus (GDM)		26	0.25	
HTN	Stage I		6,542	67.03	12,744
	Stage II		4,571	35.87	
	Severe HTN		1,603	12.57	
	Others^*^		28	0.21	
HIV/AIDS	Children (0–14) years	Regimen I	9,035	3.05	296,593
	(*n* = 10,346)	Regimen II	1,311	0.44	
	Adolescence (15–19) years	Regimen I	8,355	2.82	
	(*n* = 9,895)	Regimen II	1,540	0.52	
	Adult (20–49) years	Regimen I	210,938	71.12	
	(*n* = 222,601)	Regimen II	11,500	3.88	
		Regimen III	163	0.05	
	Adult (>50) years	Regimen I	51,988	17.53	
	(*n* = 5,3751)	Regimen II	1,728	0.58	
		Regimen III	35	0.01	

* Isolated hypertension, Regimen, Based on WHO treatment regimen HIV/AIDS.

### The trend of loss to follow-up and new treatment initiation of HIV/AIDS, DM, and hypertension during the pre-COVID-19 and COVID-19 periods

**Figure 1:**
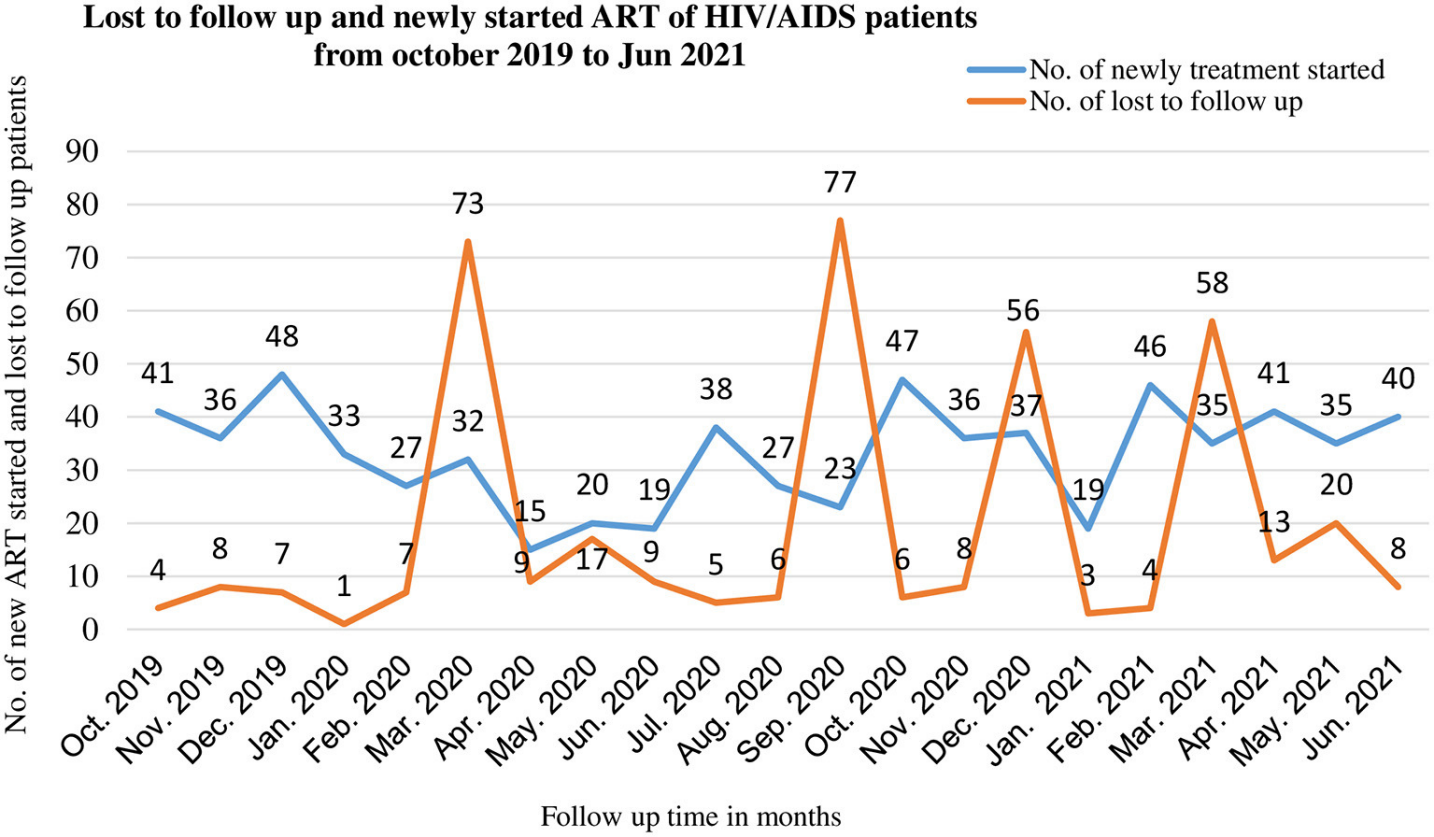
Lost to follow up and new initiation of ART among PLWHA during pre-COVID-19, and COVID-19 periods in 21 months in Northwest Ethiopia.

**Figure 2:**
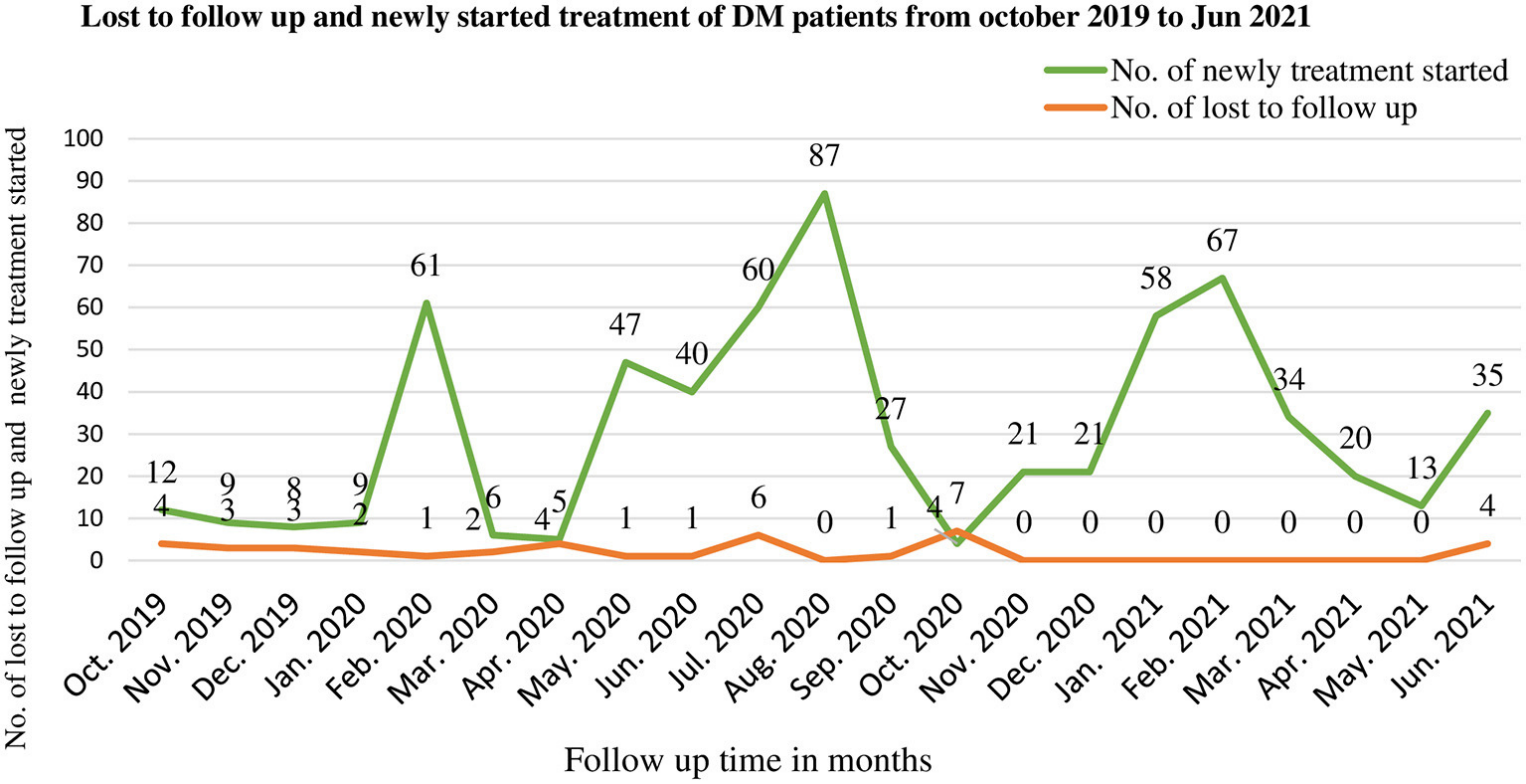
Lost to follow up and new initiation of treatment among DM patients during pre-COVID-19, and COVID-19 periods in 21 months in Northwest Ethiopia.

As seen from the following graphs the number of patients lost to follow-up from ART was low before the COVID-19 period with a maximum of 8 patients during November 2012. Whereas, during the COVID-19 period, there were multiple peaks of loss to follow up from ART, especially in March and September 2012 which were 73 and 77 respectively. The number of patients who were newly started ART was high before the COVID-19 period with a maximum of 48 patients in December 2012, but this decreased to 15 patients in April 2012 after COVID-19 entered Ethiopia. However, some improvements were seen when it becomes adopted in 2013 (Figure 1). There is no significant difference in loss to follow-up of DM patients between pre-COVID-19 and COVID-19 seasons, but there were multiple peaks in new treatment-started cases after COVID-19 entered Ethiopia (Figure 2).

**Figure 3:**
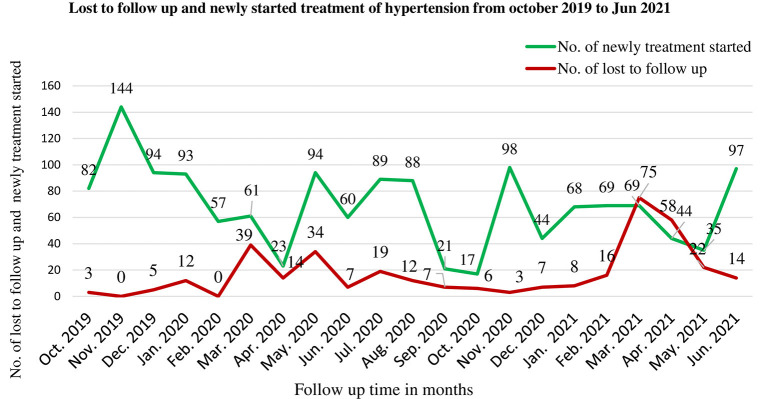
Lost to follow up and new initiation of treatment among hypertensive patients during pre-COVID-19, and COVID-19 periods in 21 months in Northwest Ethiopia.

The number of lost to follow-up of hypertensive patients was low before the COVID-19 period with a maximum of 12 patients during January 2012. Whereas, during the COVID-19 period, there were increments of loss to follow-up especially as soon as COVID-19 entered Ethiopia in March (39). The number of new patients started on antihypertensive medication was high before the COVID-19 period with a maximum of 144 patients in November 2012 but a sharp decrease to 23 in April after COVID-19 entered Ethiopia (Figure 3).

**Figure 4:**
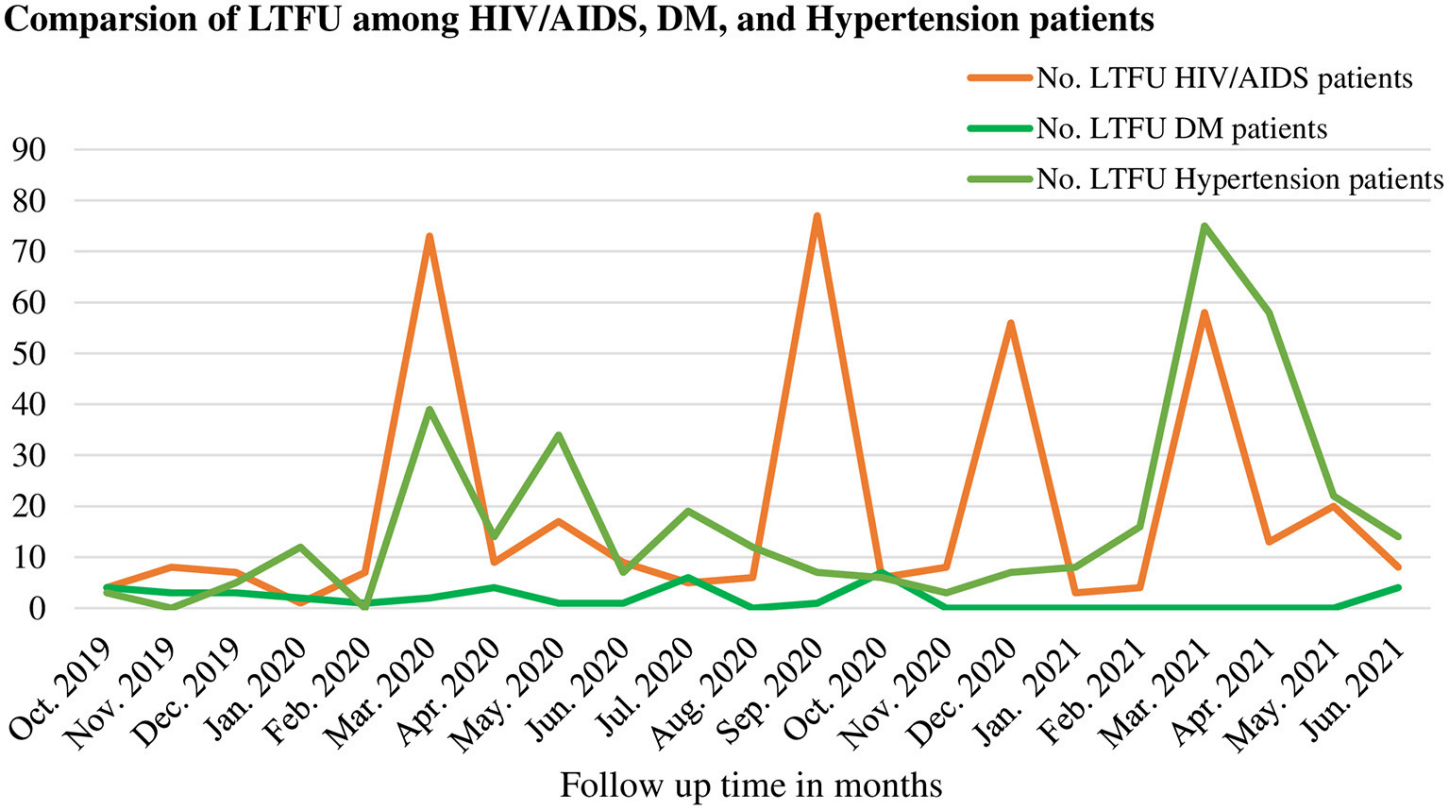
Comparisons of lost to follow up among HIV/AIDS, DM, and hypertension patients during pre-COVID-19, and COVID-19 periods in 21 months in Northwest Ethiopia.

The number of LTFU among patients during pre-COVID-19 and COVID-19 periods was compared among the three chronic diseases i.e., HIV/AIDS, DM, and hypertension patients. There was a considerable number of patients with LTFU from HIV/AIDS care during the COVID-19 period and next to that LTFU from hypertension was the most common (Figure 4).

### Mean difference analysis of loss to follow-up and new initiation of treatment among HIV/AIDS, DM, and hypertension patients during pre-COVID-19 and COVID-19 period at facilities in Northwest Ethiopia

There was a significant mean difference in the number of patients of LTFU from ART during COVID-19 and before COVID-19 with a mean difference [μd = 17.85; 95%CI: 3.25, 32.95]. There was also a significant mean difference [μd = 17.31; 95%CI: 8.35, 26.97] of patients lost to follow-up from antihypertensive treatment during the COVID-19 period as compared to the pre-COVID-19 period. But there was no significant difference in the mean number of patients with LTFU from DM treatments [μd = 0.975; 95%CI: −3.27, 1.32]. There was a significant decrease in the mean number of patients who newly started anti-hypertensive treatment during COVID-19 as compared to before COVID-19 [μd = −32.94; 95%CI: −63.76, −2.12]. But, there was no significant difference in the mean number of patients who new started ART and DM treatments during the COVID-19 and pre-COVID-19 periods [μd =14.26; 95%CI: −11.58; 40.12] (Table 2).

**Table 2:** Independent *t*-test analysis of loss to follow-up and new initiation of treatment among patients in chronic disease follow-up during COVID-19 and pre-COVID-19 periods in Northwest, Ethiopia from October 2020 to Jun 2021.

**Number of patients LTFU**	**Before COVID**−**19** **(k** = **5 months)**			**During COVID**−**19** **(k** = **16 months)**			**Mean difference**	**95%CI**	***P*** **–value**
	***n*****, mean** ± **SD**			***n*****, mean** ± **SD**					
No. of patients LTFU from ART (*N =* 399)	27	5.4	2.88	372	23.25	26.30	17.85	3.25, 32.95	0.03
No. of patients LTFU from DM (*N =* 39)	13	2.6	1.14	26	1.625	2.33	−0.975	−3.27, 1.32	0.38
No. of patients LTFU from hypertension (361)	20	4	4.95	341	21.31	20.48	17.31	8.35, 26.97	0.02
**New initiation of treatments**									
No. of patients' new initiation of ART (*N =* 695)	185	37	7.969	510	31.88	10.125	−5.12	−15.54, 5.29	0.316
No. of patients newly started treatment of DM (*N =* 644)	99	19.08	23.08	545	34.06	24.37	14.26	−11.58, 40.12	0.26
No. of patients newly started anti–hypertensive treatment (1,447)	470	94	31.678	977	61.06	27.90	−32.94	−63.76, −2.12	0.037

## Discussion

The COVID-19 pandemic has had a terrific impact on all aspects of human life and disrupted healthcare systems around the world (16). This study aimed to compare the mean loss to follow-up from chronic care services during the pre-COVID-19 and COVID-19 period from health facilities in Northwest Ethiopia.

Based on this, there was a significant mean difference in patients' LTFU from ART care during the COVID-19 period as compared to the pre-COVID-19 period. This is in line with other studies in Ethiopia that showed that COVID-19 affects Routine HIV Care Services (17). Patients are more likely to miss follow-ups during the COVID-19 pandemic period when they are above 60 years old (18). A study in Brazil and Belgium shows that a high percentage of participants reported an interruption of ART during the COVID-19 lockdown period (19, 20). A study in Italy also showed that the persistence of the COVID-19 pandemic can increase the risk of being lost to follow-up (21). Moreover, a study in Nigeria showed that there was a shortfall in the availability and a rise in the cost of ARVs during the COVID-19 pandemic (22). The reason was ART stock out at the clinic/pharmacy, inability to go collect ART due to mobility restrictions, financial constraints (19), fear of COVID-19 at the hospital, and transportation problems (18).

Moreover in this study, the mean number of patients who were new initiation of ART decreased during the COVID-19 pandemic period, but not at a significant level. This is in line with a previous study done in Ethiopia that showed a low prevalence of voluntary testing and counseling (VCT) and physician-initiating counseling and testing (PICT) during the COVID-19 pandemic period (17). Moreover, a study in Italy showed that there was a decrease in new diagnoses of HIV/AIDS (21).

This study shows that there was a significantly high mean number of patients with LTFU from hypertension treatment care during the COVID-19 period as compared to the pre-COVID-19 period. On the other hand, there was also a significant decrement in the mean number of patients who were newly started anti-hypertensive treatment care during the COVID-19 period. This might be due to that hospitals were crowded with COVID-19 patients and becomes a center of fear. On the other hand, there are depression symptoms and psychological impacts of the COVID-19 pandemic in hypertensive patients (23). Because hypertension was more prevalent among COVID-19 death in comparison with survivors (24). Hypertension and diabetes treatment with ACE2-stimulating drugs increases the risk of developing severe and fatal COVID-19 (25). These chronic illnesses also delay viral clearance in COVID-19 patients (26).

In our study, there is no significant change in the mean number of diabetics patients with LTFU from treatment care or newly started treatment during the COVID-19 period as compared to the pre-COVID-19 period. This study was supported by other studies (24, 27). The reason for our finding might be that most of the DM patients in our study were type II and were taking tablets in their homes for more than the recommended follow-up time with a self-test glucometer unlike those HIV/AIDS and/or hypertensive patients.

This finding is timely and important evidence to assess the impact of COVID-19 on chronic disease care and treatment in the study area and further insight into the country. But this study is not free of limitations. This study was conducted during the COVID-19 pandemic in Ethiopia. Therefore, for the sake of early understanding of the burden of COVID-19 in chronic disease follow-up and early identifying the gap, we declared lost follow-up from chronic care for our study, when the patient didn't take their medication for at least 1 month and above for all the three chronic diseases (HIV/AIDS, DM, and Hypertension).

## Conclusion

There was a significantly higher mean difference in the number of patients with LTFU from Anti-Retroviral Therapy (ART) and antihypertensive treatment during COVID-19 as compared to the pre-COVID-19 period. The mean number of patients who were newly started anti-hypertensive treatment during the COVID-19 season was significantly decreased as compared to the pre-COVID-19 period. Therefore the Ministry of Health Ethiopia (MOH) should be revised and modify the chronic disease follow-up guidelines and prepared a convenient follow-up method such as remote management for the ongoing COVID-19 pandemic in Ethiopia.

## Data availability statement

The data analyzed in this study is subject to the following licenses/restrictions: A national annual reportable data. Requests to access these datasets should be directed to danielgashaneh28@gmail.com.

## Ethics statement

The studies involving human participants were reviewed and approved by University of Gondar IRB. Written informed consent for participation was not required for this study in accordance with the national legislation and the institutional requirements.

## Author contributions

The conception of the work, design of the work, acquisition of data, analysis, interpretation of data, data curation, drafting of the article, critical revison for intellectual content, validation, and final approval of the version to be published was done by DB and AA. Both authors read and approved the final manuscript.

## Conflict of interest

The authors declare that the research was conducted in the absence of any commercial or financial relationships that could be construed as a potential conflict of interest.

## Publisher's note

All claims expressed in this article are solely those of the authors and do not necessarily represent those of their affiliated organizations, or those of the publisher, the editors and the reviewers. Any product that may be evaluated in this article, or claim that may be made by its manufacturer, is not guaranteed or endorsed by the publisher.

## Abbreviations

AIDS: Acquired immunodeficiency syndrome
ART: Antiretroviral treatment
CD4: Cluster of differentiations four
CI: Confidence Interval
COVID-19: Coronavirus disease 2019
HIV: Human Immunodeficiency Virus
LTFU: Lost to follow up
PLWH: People living with HIV/AIDS
MOH: Ministry of Health Ethiopia.
